# Supplementary material for: A UMLS-based spell checker for natural language processing in vaccine safety
Source: BMC Med Inform Decis Mak. 2007 Feb 12;7:3. doi: 10.1186/1472-6947-7-3 (PMC1805499; doi:10.1186/1472-6947-7-3)
Supplement: Additional file 13 — RAP application directory. Sets up directory for RAP (RDF application for PHP) source code [file 1472-6947-7-3-S13.gz › rap/doc/phpdoc/__filesource/fsource_resModel__resModelResBag.php.html]

xml version="1.0" encoding="iso-8859-1"?


File Source for ResBag.php


# Source for file ResBag.php

Documentation is available at ResBag.php

```
1. <?PHP
2. // ----------------------------------------------------------------------------------
3. // Class: ResBag
4. // ----------------------------------------------------------------------------------


8. /**
9. * This interface defines methods for accessing RDF Bag resources.
10. * These methods operate on the RDF statements contained in a model.
11. *
12. * <BR><BR>History:<UL>
13. * <LI>10-01-2004                : First version of this class.</LI>
14. *
15. * @version  V0.9.1
16. * @author Daniel Westphal <mail at d-westphal dot de>
17. *
18. * @package     resModel
19. * @access    public
20. ***/
21. class ResBag extends ResContainer
22. {

24. /**
25. * Constructor
26. * You can supply a URI
27. *
28. * @param string $uri
29. * @access    public
30. */
31. function ResBag($uri = null)
32. {
33. parent::ResContainer($uri);
34. $this->containerType=new ResResource(RDF_NAMESPACE_URI.RDF_BAG);
35. }
36. }
37. ?>
```

Documentation generated on Thu, 7 Jul 2005 13:43:30 +0200 by phpDocumentor 1.3.0RC3
